# Interprofessional collaboration and smartphone use as promising strategies to improve prenatal oral health care utilization among US underserved women: results from a qualitative study

**DOI:** 10.1186/s12903-020-01327-9

**Published:** 2020-11-23

**Authors:** Lin Wang, Johana Ren, Kevin A. Fiscella, Sherita Bullock, Mechelle R. Sanders, Elizabeth L. Loomis, Eli Eliav, Michael Mendoza, Rita Cacciato, Marie Thomas, Dorota T. Kopycka-Kedzierawski, Ronald J. Billings, Jin Xiao

**Affiliations:** 1 Eastman Institute for Oral Health, University of Rochester Medical Center, Rochester, NY USA; 2 Peking University School of Stomatology, Beijing, China; 3 University of Rochester River Campus, Rochester, NY USA; 4 Department of Family Medicine, University of Rochester Medical Center, Rochester, NY USA; 5 Healthy Baby Network, Rochester, NY USA; 6 Monroe County Department of Public Health, Rochester, NY USA

**Keywords:** Prenatal oral health, Underserved pregnant women, Inter-professional collaboration

## Abstract

**Background:**

Data on barriers and facilitators to prenatal oral health care among low-income US women are lacking. The objective of this study was to understand barriers/facilitators and patient-centered mitigation strategies related to the use of prenatal oral health care among underserved US women.

**Methods:**

We used community-based participatory research to conduct two focus groups with eight pregnant/parenting women; ten individual in-depth interviews with medical providers, dental providers and community/social workers; and one community engagement studio with five representative community stakeholders in 2018–2019. Using an interpretive description research design, we conducted semi-structured interviews and focus groups which were audio-recorded, transcribed, and analyzed for thematic content.

**Results:**

We identified individual and systemic barriers/facilitators to the utilization of prenatal oral health care by underserved US women. Strategies reported to improve utilization included healthcare system-wide changes to promote inter-professional collaborations, innovative educational programs to improve dissemination and implementation of prenatal oral health care guidelines, and specialized dental facilities providing prenatal oral health care to underserved women. Moreover, smartphones have the potential to be an innovative entry point to promote utilization of prenatal oral care at the individual level.

**Conclusions:**

Low-income women face multiple, addressable barriers to obtaining oral health care during pregnancy. Inter-professional collaboration holds strong promise for improving prenatal oral health care utilization.

## Background

Despite the well-documented association between poor maternal oral health, preterm/low-birth-weight deliveries [1, 2] and increased tooth decay in children after birth [3], many mothers-to-be do not receive timely oral health care [4]. Oral health is an important component of general health and should be maintained during pregnancy [5]. Although recommendations for prenatal oral health care have been widely disseminated in the US at both the state [6] and national level [7], utilization of prenatal oral health care remains low. A national survey [8] recently released by a dental insurance company, Cigna, found that 43% of women did not have a dental checkup during pregnancy while 76% admitted to suffering from oral health problems (pain, gum bleeding and oral infection) during pregnancy. Oral health care utilization during pregnancy is even lower among African American women [9], ethnic minorities [10], and women with socioeconomic impediments [11]. Thus, prenatal oral health represents an important, but often-neglected health disparity [12, 13]. Understanding factors that enable or hinder the use of prenatal oral health care is crucial to identify effective strategies to promote and maintain good oral health in pregnant women.

At the patient level, demographic, socioeconomic, psychological, and behavioral factors affect utilization of prenatal dental care. Among women living in California, a population-based study found that the primary reason for not obtaining dental care was lack of perceived need, followed by financial barriers [4]. A survey among 625 women (a predominantly Caucasian population of relatively high socioeconomic status) living in Johnson County, Iowa, showed that increased prenatal dental visits were associated with being married, having more frequent dental visits when not pregnant, having dental insurance, and having knowledge of the possible connection between oral health and pregnancy outcomes [14]. Similar effects have been reported globally. Australian [15, 16] and Iranian pregnant women [17] reported that barriers to utilization of prenatal dental care included lack of knowledge, false beliefs, cost, fear, the limited number of dentists providing care to pregnant women, and inadequate recommendations from health professionals.

At the clinician level, recommending prenatal oral health care utilization is not routinely practiced by maternity providers (general practitioners, obstetricians, and midwives) [18, 19]. A national survey [20] among 366 US primary care physicians (PCPs) reported that 37% of PCPs rarely/never provided oral health counseling to their pregnant patients. Being a female PCP, receiving continuing education on oral health-related topics, perceived preparedness to provide oral health counseling, and counseling adults with other health conditions contributed to increased prenatal oral health counseling [20]. Moreover, midwives in Australia reported a reluctance to discuss oral health with pregnant women and an unawareness of the possible impact of poor oral health on maternal and child health, as well as not knowing appropriate referral pathways to Public Dental Services [21].

Data on barriers and facilitators to prenatal oral health care among low-income US women are lacking. Given differences in cultural beliefs, social environments, and health care systems, it is challenging to apply findings from other populations to women receiving care in underserved settings in the US. To address this gap, we used community-based participatory research (CBPR) [22] to assess perceptions, barriers and facilitators, and patient-centered mitigation strategies related to use of prenatal oral health care by underserved US women and by a cohort of medical/dental providers and community/social workers. Qualitative analysis of data used an interpretive description research design with triangulation across multiple data sources.

## Methods

### Study design

We examined a clinical phenomenon (limited utilization of prenatal oral health care) to identify themes and patterns related to reported perceptions of oral health care among underserved women, medical/dental providers and community/social workers. We used the interpretive description [23] research design which is best suited to investigate problems that are rooted in clinical practice. This design was chosen to develop a coherent professional narrative that informs clinical practice and promotes a practice or policy change. Throughout the study, we applied the principle of respect, reactivity, and reflexivity to build a trusting relationship between study participants and study personnel, based on the strategies of Paterson [24]. This study was approved by the University of Rochester Medical Center Research Subjects Review Board (RSRB 72596). We included all points in a checklist of COREQ guidelines [25] for reporting focus groups and interviews. The items for COREQ domain 1 are listed in "Appendix 1".

### Research settings and procedure

#### Study site

This study was conducted in 2018–2019 in Rochester, NY. Rochester is the third most populous city in New York State, with a population of 208,046 residents. The University of Rochester Medical Center and Rochester Regional Health System are the two leading providers of comprehensive care for Rochester residents holding both private and state-supported medical insurance. Pregnant women whose income is equal to or lower than 138% of the Federal Poverty Threshold are eligible for NY State-supported dental insurance (Medicaid) which provides coverage for adult dental procedures including comprehensive oral examinations, extractions, and restorative/endodontic/periodontal procedures [26].

We collaborated with two local organizations (University of Rochester and Healthy Baby Network) on the development of the study design, participant recruitment, and data collection and interpretation. The University of Rochester provides obstetric services to more than 4000 pregnant women per year (approximately 40% of women are African American, 40% Caucasian, and 20% other). Three-quarters of these pregnant women benefit from state-supported dental insurance that is provided to low-income groups (Medicaid). The Healthy Baby Network is a Rochester based non-profit social service organization dedicated to improving the health and well-being of mothers and babies by addressing systemic barriers that contribute to racial health disparities and that adversely impact healthy births. The Healthy Baby Network provides outreach services to over 200 low-income pregnant women per year.

#### Eligibility and recruitment

All study participants were 18 years of age or older. Five types of informants were included. Pregnant or parenting women (first group of informants) who participated needed to be pregnant or have at least one child < 2 years of age, be eligible for state-supported insurance (e.g. Medicaid, Blue Choice, MVP Option). Medical providers (second group of informants), dental providers (third group of informants) and community/social workers (fourth group of informants) who participated in the study were required to be currently practicing. The fifth group of informants were community stakeholders purposefully selected by the community studio organizer (see below).

Subjects who had decisional impairment or were incapable of making an informed decision about participation in the study were excluded. Recruitment was conducted face-to-face by two trained research coordinators, and study participants were consented prior to the beginning of study activities. Community stakeholders did not require formal consent in accordance with standard community studio practice.

#### Focus group discussion

Two 2-h focus group sessions were conducted with eight pregnant or parenting women using a semi-structured interview guide ("Appendix 2"). The interview guide was pilot-tested by three low-income women prior to launching the study. We asked the women about their experiences and perceptions about obtaining oral health care during pregnancy, and perceptions about women’s oral health. The focus group discussions were conducted in private rooms, at locations convenient for participants. A research assistant took notes and audio recorded each focus group session.

#### Individual interview

Ten 60-min semi-structured individual interviews were conducted by a trained facilitator and included four community/social workers, three medical providers and three dental providers. Using a pilot-tested semi-structured interview guide ("Appendix 2"), we asked questions that addressed perceptions about barriers and facilitators that were associated with utilizing prenatal oral health care, including perceptions about promoting women’s oral health. The interviews were conducted in private rooms with a research assistant who took notes and made audio recordings.

#### Community engagement studio

Following the completion of focus groups, individual interviews, and initial data analysis, we conducted a 2-h Community Studio among five representatives of community stakeholders to gather their opinions on our initial summary of barriers and facilitators to prenatal utilization of oral health care. The Community Studio method, developed at Vanderbilt, was chosen to assemble community representatives to provide input into research data interpretation and analysis [27]. Community stakeholders’ suggestions gathered at the Community Studio were elicited and incorporated into the final data analysis.

#### Rigor and validity

We used the following methods to promote validity: (1) triangulation: we used different data collection methods detailed in the data collection methods, including individual interviews, focus groups, field notes and memos; (2) we applied principle of respect, reactivity [24], reflexivity to build a trusting relationship between study participants, study interviewers, and focus group facilitators. This method was used for interviews and group discussion questions and throughout all aspects of interactions between participants and study team members.

Importantly, we adopted the following strategies from Paterson [24]: (1) express empathy: during the research process, team members showed empathy with participants to minimize the “stress of entry” and to encourage study participants to be more expressive in talking about the study topic; (2) empower study participants: distribute power to study participants, such that study participants have authority over their own oral health and autonomy during the interview; (3) respect cultural differences: study team members listened more to participants’ opinions and cultural attitudes about oral health without judgment.

### Data analysis

Audio recordings were initially transcribed by the Temi (USA) transcription service, and further verified by two trained researchers. Transcribed data were stored and analyzed using MAXQDA software (VERBI GmbH, Berlin, Germany). The data were coded by two trained coders with predetermined and later modified (during data analysis) open codes using a codebook with a description of the coding tree ("Appendix 3"). Thematic content was further analyzed using categorizing and contextualizing strategies to understand the factors associated with utilization of prenatal oral health care among underserved women.

## Results

### Sample

The focus group of pregnant and parenting women were 27–32 years of age, with a mean age of 28.4 years. The individual interviews were completed with four social workers, three medical providers (one PCP and two OBGYNs), and three dental providers (one hygienist and two community dentists). All participants were female, 34–63 years of age, with a mean age of 47.9 years. We did not encounter participants who refused to join the study or who dropped out. We reached data saturation by the end of the study, and after we were no longer receiving new information from additional interviewees.

### Women’s experiences of oral health problems during pregnancy

The focus group of pregnant and parenting women repeatedly reported experiencing dental problems during pregnancy. Many reported not knowing the importance of maintaining good oral health during pregnancy and described seeking dental care during pregnancy as “frustrating” and “did not know it is needed.” For example,

parenting woman: “I had issues with my gums, probably at mid pregnancy, it started swelling, bled and hurt. But I didn't go to the dentist. I just kind of dealt with it.”

### Barriers and facilitators

Comments from all five types of informants were categorized as barriers, facilitators, and strategies or implications for policy-making. Table 1 illustrates representative quotations. Table 2 summarizes themes from our analysis of participant comments, separated into identified individual and system-level factors.

**Table 1:** Illustrative quotations from study informants: barriers and facilitators to prenatal oral health care utilization

Barriers	Informants	Illustrative quotations
Socioeconomic hardships and competing interests	Parenting woman	“I'm not so pressured to figure out dental care for myself, at least not when I have other things going on that's more important than dental care”
Lack of awareness of benefits and importance	Parenting woman	“I wish I knew more about that my dental health has something to do with my baby’s health”
Lack of awareness of dental coverage from medical insurance	Social worker	“The population we serve has Medicaid insurance which is comprehensive. But I'm not sure that it's clear to the patients that it includes dental coverage”
Inadequate inter-professional collaboration	Parenting woman	“My provider never told me that was a problem. I started having bleeding gums and then I looked it up online. That's how I know about it”
	Parenting woman	“It would be good if the OB would address this. Have you seen your dentist? That would be one way to make pregnant women go see the dentist”
Lack of awareness of prenatal oral health guidelines	DDS/DMD	“Some patients worry about dental treatment during pregnancy. Currently, we just give patient verbal education, no handouts, we just tell patient due to the hormone change, you need to have better oral hygiene. If there is a guideline that we can show them, it is better”
	OBGYN	“In medical school, we didn’t have classes for oral health. None”
Insufficient dentists providing treatment to underserved pregnant women	Parenting woman	“The worst thing of being pregnant is you have toothache. So I'm just going to be honest with you. I had a bad tooth, I was in a lot of pain, but they (dentists) would not pull it because I was pregnant. I had to go to another doctor, acted like I wasn't pregnant and they pulled the tooth out”
	OBGYN	“Oftentimes what comes into play is no one (dentists) will see them (pregnant women). They (patients) told me, they are in too much pain, but the dentists won't do anything, they will ask me can you do something?”
Facilitators	Informants	Illustrative quotations
Constant reminders	Parenting woman	“I need constant reminders by my OB. When I find out I'm pregnant, that could be a good time to be asked, hey, do you know this will cause problem to your baby? Then, when I go to my next appointment, they ask me, hey, here's some more information about this, the germs in your mouth could be passed to your baby. When I go to my next appointment, I will be asked again, hey, I don't know if you took a look at that paperwork? A constant reminder goes a long way”
Raise community awareness via mass media	Parenting woman	“It (prenatal oral health education) has to be interesting, I would say more of a commercial on TV. Whether I'm eating, or I'm talking on the phone, my eyes are still on TV. I can read what's on the TV, that's going to be in my mind. Even if I don't hear nothing about what they're saying, I'm, at least, going to remember something from that commercial”
Strengthen interprofessional collaboration	OBGYN	“Adding an additional task to an already packed prenatal schedule for medical providers can be challenging. But I think our midwifery colleagues are a bit flexible and they often do some more holistic family care. For example, they make home visits in the neonatal period. Introducing the idea of oral health in that community might be reinforcing”
	PCP	“The system for EPIC, I do not think is yet to set up having special health maintenance tabs for pregnancy. But if there was, you could have a health maintenance checklist that says, dental care is overdue or is unsatisfied at this time. That would be a reasonable thing to consider for all pregnant patients”
	OBGYN	“OBGYN, family doctors or pediatricians giving oral health education, potentially, depends on what is involved to satisfy that billing code”
	DDS/DMD	“The social workers are also very important for spreading correct oral health knowledge for pregnant women. They can work as a bridge to help mothers getting sufficient dental education and dental care in community”
	OBGYN	“There is a push currently to offer doula services to a broader range of patients. I think expand Medicaid coverage to cover doulas for lower income populations, who are oftentimes going out to the community to support. So that would be another place to introduce oral health education during pregnancy”

**Table 2:** Summary of barriers, facilitators, strategies and implacations for policy-making

Factors	Barriers	Facilitators	Strategies and implications for policy-making
Individual level	Socioeconimic hardships lack of babysitting lack of transportation	Receive “constant” reminders [actual reminders from health care navigators and virtual reminders from mobile phone device (e.g., texting, smartphone app)	Use social media and smartphone device to promote prenatal oral health education and oral health care utilization
	Competing interest		
	Lack of awarenss of benefits and importance	Receive prenatal oral health education and dental resources information on clinic resources and insurance coverage through smartphone device (social media, app)	
	Lack of awarenss of dental coverage from state-supported medical insurance	Community-wide dissemination via mass media (e.g., TV commercials) of the benefits and importance of prenatal oral health and dental insurance coverage	
System level	Inadequate inter-professional collaboration	Advocacy by medical providers and social/community workers about the importance of prenatal oral health	Create healthcare system-wide change to promote interprofessional collaborations - Incorporate dental care into the medical care setting: shared physical location and shared electronic record system - Promote prenatal oral health counseling by non-MDs, e.g., nurse practitioners, midwives, medical technicians, and social workers. Billable prenatal oral health counseling service provided by medical providers and staff - Improve collaborations with social benefit programs (e.g., WIC), use innovative mediators to promote prenatal oral health, e.g., Doula, peer councilors
	Lack of awarenss of latest prenatal oral health guidelines		Introduce innovative educational program to improve prenatal oral health care guidelines dissemination and implementation among medical/dental providers and community/social workers
	Insufficient dentists providing treatment to underserved pregnant women		Develop specialized dental facilities providing prenatal oral health care to underserved groups, Hub-Spoke model

#### Individual-level barrier theme 1: socioeconomic hardships and competing interests

A commonly recognized barrier to medical care utilization, e.g., socioeconomic hardships and competing interests, also represented a barrier to utilization of prenatal oral health care***. ***Pregnant/parenting women reported that they could not get to their dental appointments due to lack of babysitting, lack of transportation, and priority of other life events.

#### Individual-level barrier theme 2: lack of awareness of benefits and importance

Another barrier was unawareness of the benefits and importance of prenatal oral health care among pregnant women. Many pregnant/parenting women reported lack of knowledge about the association between poor oral health and adverse birth outcomes, and the importance of maintaining good oral health during pregnancy.

#### Individual-level barrier theme 3: lack of awareness of dental coverage from medical insurance

A unique barrier identified was unawareness of having dental coverage as a recipient of state provided medical insurance, e.g., Medicaid. A majority of medical providers, community/social workers, and pregnant/parenting women did not know about dental benefits associate with Medicaid. In addition, they were unaware of dental clinics that accepted Medicaid.

#### System-level barrier theme 1: inadequate inter-professional collaboration

Intriguingly, all participating medical providers, dental providers, community/social workers, and pregnant/parenting women recognized insufficient inter-professional collaboration in promoting prenatal oral health. A majority of informants felt that the initial introduction about the importance of prenatal oral health should be initiated by medical providers.

#### System-level barrier theme 2: lack of awareness of prenatal oral health guidelines

We identified another system-level barrier: unawareness of the latest practice guidelines among medical and dental providers, and among community/social workers. Although some of them knew a few prenatal oral health guidelines, none of them were aware of the latest practice guidelines, including recommendations from professional organizations, such as the American College of Obstetricians and Gynecologists or the American Dental Association, both of which clearly state conditions that require immediate treatment, such as oral examinations, extractions, root canal treatment, and fillings are important and safe to perform at any time during pregnancy [28]. Some OBGYNs said they continued to receive requests from dental providers of their pregnant patients for medical clearance to initiate dental treatment. This unfamiliarity with the practice guidelines was echoed by other participating dentists, who reported avoiding providing dental treatment to pregnant women during their 1st trimester, although the ACOG and ADA recommend the 1st trimester as a safe period for receiving dental care.

#### System-level barrier theme 3: insufficient dentists providing treatment to underserved pregnant women

Both medical providers and community/social workers reported that they had referred their pregnant patients for oral health care but, unfortunately, some of their patients were denied dental care due to the pregnancy. Furthermore, the pregnant/parenting women expressed the notion that only a limited number of dental clinics accepted their State-supported insurance (e.g., Medicaid) and the long waiting list for care exacerbated their unwillingness to visit a dentist during pregnancy.

### Facilitators

#### Individual-level facilitator theme 1: constant reminders

A critical facilitator that pregnant/parenting women proposed was “constant reminders.” Participating women stressed that these reminders were important and should be introduced by their maternity health care team as early as their first pregnancy visit, and repeated at their follow-up pregnancy appointments.

#### Individual-level facilitator theme 2: raise community awareness via mass media

Pregnant/parenting women suggested raising community awareness via mass media as an essential facilitator to improve the use of prenatal oral health care. They recommended the use of commercials, informational campaigns, bus stop banners, health fairs, and community events.

#### System-level facilitator theme 1: strengthen interprofessional collaboration

Strengthening interprofessional collaboration was proposed as a strong facilitator to promote the use of prenatal oral health care. Medical and dental providers, and community/social workers specifically commented on several pivotal points that are crucial to a sustainable interprofessional collaboration, including merging the medical and dental electronic record systems, shortening physical distance between prenatal medical and dental offices, creating billable prenatal oral health counseling services, and enhancing collaborations with social service groups.

### Innovative entry points for prenatal oral health care promotion

**Fig. 1:**
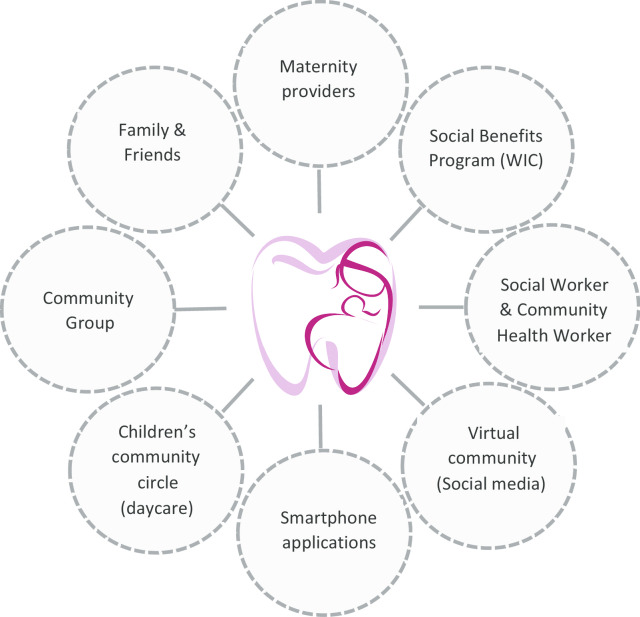
Entry point for promoting prenatal oral health care. WIC, Women, Infants and Children, is a federal assistance program of the Food and Nutrition Service (FNS) of the United States Department of Agriculture (USDA) for healthcare and nutrition of low-income pregnant women, breastfeeding women, and children under the age of five

Through this study, we further mapped the elements of both the real and virtual communities that women interacted with during pregnancy; these are demonstrated in Fig. 1. The map reveals the potential entry points that can promote the importance and benefit of prenatal oral health care to the majority of underserved pregnant women. These entry points include traditional medical contacts through maternal providers; traditional social circles such as community groups, family and friends; social benefit support groups; children’s community circles (e.g. daycare), and social workers. What stands out as an innovative entry point is the virtual community, through which social media and smartphone apps can disseminate information, as illustrated in the quotes below.

Parenting woman: “Nowadays, most people will use smartphones. Smartphones will be a good way for patients to get oral health information. For example, if they are pregnant or breastfeeding, they can download an app with a professional dental knowledge plugin at every different stage. When they are reviewing pregnancy or breastfeeding related information, noticeable pictures and videos will provide better oral health education, not only for moms, but also for young children.”

Parenting woman: “It should be a commercial somewhere, whether it's Facebook and it could be played between a popular show that everyone is watching.”

OBGYN: “Many of our moms are members of online support groups. I think Text-for-Baby is one that connects moms with knowledge they need to know during pregnancy.”

## Discussion

In recent years, federal agencies and professional organizations have mounted a substantial effort to address an increasingly recognized public health problem, poor prenatal oral health, which has serious implications for maternal and child health. Yet, prenatal oral health care is significantly underutilized in the US (and worldwide), especially among underserved groups, many of whom lack access to care.

This study used a community based participatory research (CBPR) strategy to engage a hard-to-reach, priority population of underserved pregnant women, and clarified barriers and facilitators to their use of prenatal oral health care. We applied the principle of respect, reactivity, and reflexivity to build a trusting relationship between study participants and study personnel. In addition, we used rigorous qualitative methods to promote validity, in particular triangulation across different data collection methods and different stakeholders. Finally, we identified barriers and facilitators that led to the identification of several innovative strategies and indications for potential policy-making regarding prenatal utilization of oral health care in the community.

Based on our study, we share below four recommendations that have potential to improve prenatal oral health care utilization in underserved US women.

1. Create healthcare system-wide changes to promote inter-professional collaboration
  *Incorporate dental care into the medical care setting*. To address the historical problem of inadequate communication between medical and dental providers, collaboration could be facilitated through shared facilities and particularly electronic record systems. Referrals, reminders, and feedback that could be easily communicated on a shared platform would significantly improve the rate of follow-through for recommended prenatal oral health care.
  *Promote prenatal oral health counseling by non-MDs.* Within the scope of prenatal oral health, the professionals who could make significant impactful changes include non-MD providers, e.g., midwives [29], nurse practitioners, the Centering Pregnancy Prenatal Care groups [30] and medical technicians. To maintain sustainability, billable prenatal oral health counseling services provided by non-dental providers should be pursued.
  *Improve collaboration with social benefit programs*, e.g., Women, Infant and Children (WIC). Use innovative mediators to promote prenatal oral health, such as community/social workers, peer counselors and doulas. In Klamath County, Oregon, Milgrom et al. [31] demonstrated a successful and sustainable prenatal oral health care model by providing home/WIC visits to low-income pregnant women within a dental managed care program, setting an example for successful collaboration with social benefits programs in promoting prenatal oral health.
2. Introduce innovative educational programs to improve dissemination and implementation of prenatal oral health care guidelines
  A majority of the medical/dental providers and community social worker participants were unaware of the current prenatal oral health care guidelines, therefore the first step should be to increase the awareness of guidelines. Lee et al. [32] investigated to what extent dentists adopted perinatal care practice guidelines and found that correct knowledge about the appropriateness of routine services and emergency procedures for pregnant patients is essential for adherence to practice guidelines. The second step is to identify guidelines implementation strategies to change practice patterns. As Bahrami et al. [33] noted, existing implementation strategies are not panaceas and few studies have investigated their effectiveness in dentistry. Innovative continuing education programs are critically needed to address this gap*.*
3. Develop specialized dental facilities to provide prenatal oral health care to underserved groups.
  Use a Hub-Spoke model; highly specialized dental facilities that are dedicated to underserved pregnant women could serve as the hub to meet regional patients’ needs and educate staff at satellite facilities. The University of Rochester Eastman Institute for Oral Health (EIOH) has robust clinical services with a strong community orientation. The most significant recent change to the current prenatal oral care services in Rochester has been the new EIOH Pregnancy and Infant Dental clinic, which is dedicated to underserved pregnant women and their infants.
4. Use social media and smartphones to promote prenatal oral health education and oral health care utilization.
  Smartphone health care products have been successfully applied to manage individual behaviors and health conditions [34], such as smoking cessation, weight loss, medication adherence, and Parkinson’s disease progression monitoring [34, 35] to name a few. With 81% of Americans owning a smartphone [36], a smartphone app offers a potentially high impact approach to encourage prenatal oral health care. Although there are a plethora of apps available to help expectant mothers anticipate and manage the physical and mental effects of pregnancy, none are available that specifically address pregnancy and oral health. Such an app has substantial potential to help in educating pregnant mothers and engaging them in the management of their own oral health and hygiene, as well as providing a critically needed source of information related to identifying dental clinics that provide prenatal oral health care to expectant women and in sourcing the availability of, and eligibility for, state supported dental insurance, e.g., Medicaid.

Despite the strengths of this study, our findings should to be cautiously interpreted. First, all study participants, including medical and dental providers, and community/social workers were female, which precludes generalization of our findings to male clinicians or community/social workers. Second, the study was conducted in only one city. In the US, despite the extended Medicaid health insurance coverage for pregnant women through public programs, adult dental benefits are optional for states to offer among Medicaid services. In 2014, seven states provided no dental benefits and only 17 states provided comprehensive adult dental benefits, including New York State [37]. As of 2019, dental coverage was provided to pregnant women by some US states through Medicaid; examples of the dental procedures covered are comprehensive oral examinations (31 states), adult dental prophylaxis (30 states), fluoride varnish applications (12 states), composite fillings (29 states), endodontic treatment of anterior teeth (22 states), and scaling and root planning (26 states) [38]. Although insurance coverage was not identified as a barrier in our study, low-income pregnant women without dental insurance living in other states could face a major barrier to receiving prenatal oral health care.

## Conclusions

Low-income women face multiple, addressable barriers to obtaining oral health care during pregnancy. Inter-professional collaboration holds strong promise for improving prenatal oral health care utilization. System-wide policy changes to improve such inter-professional collaboration should include explicit recommendations for prenatal oral health care from obstetrical/medical providers, effective mediation by community/social workers, and implementation of recommended universal practice guidelines for community dental providers. Furthermore, use of smartphones and social media offers an innovative entry point to promote prenatal oral care utilization at the individual level. We plan to test the effectiveness of these innovative strategies (e.g., providing personalized prenatal oral health education and oral disease remote detection via smartphones) in our future studies. The CBPR process has strengthened local research capacity and has formed ongoing relationships among study investigators, local liaisons, and the community that will be essential for the next phase of program design and policy development for future implementation.

## Data Availability

The datasets generated and/or analyzed during the current study are not publicly available due to individual privacy (HIPPA requirements) but are available from the corresponding author on reasonable request, albeit with private identifiers removed.

## Appendix 1: Consolidated criteria for reporting qualitative studies (COREQ): 32-item checklist

Domain 1: Research team and reflexivity personal characteristics.

**Table:**

No	Item	Description of the study
*Personal characteristics*		
1	Interviewer/Facilitator	Individual interview: Jin Xiao, Lin Wang Focus Group: Mechelle R Sanders (primary facilitator), Jin Xiao (secondary facilitator)
2	Credentials	Ling Wang (Female), DDS, MS, PhD, Assistant Professor Johana Ren (Female), Undergraduate student Kevin A Fiscella (Male), MD, MPH, Professor Sherita Bullock (Female), CEO of the Healthy Baby Network Mechelle R Sanders (Female), PhD, Instructor Elizabeth L Loomis (Female), MD, Assistant Professor Eli Eliav (Male), DDS, PhD, Professor Michael Mendoza (Male), MD, MPH, MBA, commissioner of the Monroe County Public Health Rita Cacciato (Female), MS, Study coordinator Marie Thomas (Female), Study coordinator Dorota T Kopycka-Kedzierawski (Female), DDS, MPH, Professor Ronald J Billings (Male), DDS, Professor Jin Xiao (Female), DDS, MS, PhD, Associate Professor
3	Occupation	
4	Gender	
5	Experience and training	
*Relationship with participants*		
6	Relation established	No prior relationship established prior to study commencement
7	Participant knowledge of the interviewer	Participants were aware of the reasoning of conducting the research during the recruitment introduction by the study coordinators
8	Interviewer characteristics	Authors adopted the following strategies from Paterson (express empathy, empower study participants, respect culture difference) while conducting the study

## Appendix 2: Interview guide for pregnant women and mothers with young children

*Experiences of using dental services*

- Have you ever visited a dentist? Where was it? How did you hear about the service and that location?
- Do you go to the dentist for check-ups or to have treatment?
- How did you get there? Did you use public transport, walk, drive, someone drove you in a car?
- Have you had any problems getting a dental appointment or getting to a dental service when you needed to?
- Have you taken your children to the dentist? Why/why not? When do you think children should see a dentist?

*Perception of oral health during pregnancy*

- Can you recall any issues you had with your teeth or gums since you were pregnant? Such as toothache, gum bleeding, or swelling in your mouth?
- Have you used a dental service when you were pregnant?
- Could you please tell me what you know about oral health?
- Have you heard that there might be associations between mother’s oral health and children’s oral health?

*Acquisition of oral health information*

- Who have talked about oral health with you in the past?
- Where would you go, or who would you ask, if you need dental information for you or your family?
- What do you think is the best way to find out about how to keep your teeth healthy? When is the best time to give you dental information?

*Smart phone application use*

- Are you familiar with smart phone application?
- Do you know any smart phone application related to your health during pregnancy?
- Will you use smart phone application to look for oral health information if there is one?
- What kind of information will you like to see on smart phone application regarding oral health, video, blog, pictures?
- Do you have any suggestions for using smart phone to promote oral health?

*WIC service use*

- Have you heard about or used WIC service? If yes, did anyone at WIC ask you about if you/your child had routine dental visits or had dental issue? How did you feel about that?

*Suggestions from the audience*

- Keep mom’s teeth healthy are important to moms’ and children’s oral and overall health, but not many pregnant moms are seeing dentists. Your children should have at least 1 dental visit by 1 year? Could you give us suggestions how we can improve that?

## Appendix 2: Interview guide for community/social workers, medical or dental providers

### Part 1 Interview guide: community/social workers sector

*Knowledge of local dental services*

- Do you provide support to underserved pregnant women?
- Do you refer women to dental services? Where? Do you think women attend? If not, what are the reasons why? Do you follow up on the outcome of referrals?
- What you know about oral health care for pregnant women?
- What obstacles are inhibiting underserved pregnant women from attending dental visits?

*Relationship between poor maternal oral health and child oral health outcomes*

- Do you know if maternal oral health is covered in Medicaid insurance? Please explain.
- Are you aware of any protocol, evidence, clinical guidelines around the relationship between maternal oral health and child oral health outcomes?
- Have you noticed that pregnant women, have specific needs regarding their dental care? How do you respond to this? How do women respond to you providing dental information?

*Service strengthening*

- How do you feel about the support you receive for providing dental advice to underserved women?
- What would you need to feel confident in providing oral health advice to pregnant women? What sort of training, practice and resources would be helpful to you?
- Do you know anyone from the dental service? Would it be useful to meet anyone? To learn how their service works?
- Do you think it’s important to create/strengthen relationships between maternity and dental services? How could this happen? Who would be important people to involve?
- Are there any organizational changes that you think might help us to improve the care and outcomes for this group?

*Smart phone application use*

- Are you familiar with smart phone application?
- Do you know any smart phone application related to patients’ health during pregnancy?
- Will you use smart phone application to look for oral health information if there is one?
- What kind of information will you like to see on smart phone application regarding oral health, video, blog, pictures?
- What do you think could be the drawback using smart phone application to deliver oral health information?
- Do you have any suggestions for using smart phone to promote oral health?

### Part 2. Interview guide: medical providers sector

*Knowledge of local dental services*

- Do you provide care to underserved pregnant women?
- Do you refer women to dental services? Where? Do you think women attend? If not, what are the reasons why? Do you follow up on the outcome of referrals?
- What you know about oral health care for pregnant women?

*Relationship between poor maternal oral health and child oral health outcomes*

- Do you know if maternal oral health is covered in Medicaid insurance in NY? Please explain.
- Are you aware of any protocol, evidence, clinical guidelines around the relationship between maternal oral health and child oral health outcomes?
- Have you noticed that pregnant women, have specific needs regarding their dental care? How do you respond to this? How do women respond to you providing dental information?

*Service strengthening*

- How do you feel about the support you receive for providing dental advice to underserved women?
- What would you need to feel comfortable in providing oral health advice to pregnant women? What sort of training, practice and resources would be helpful to you?
- Do you know anyone from the dental service? Would it be useful to meet anyone? To learn how their service works?
- Do you think it’s important to create/strengthen relationships between maternity and dental services? How could this happen? Who would be important people to involve? Would guidelines/protocols/referral pathways be useful ways of establishing this?
- Are there any organizational changes that you think might help us to improve the care and outcomes for this group?

*Smart phone application use*

- Are you familiar with smart phone application?
- Do you know any smart phone application related to patients’ health during pregnancy?
- Will you use smart phone application to look for oral health information if there is one?
- *What do you think using smart phone application to deliver education and updates to health care providers?*
- What kind of information will you like to see on smart phone application regarding oral health, video, blog, pictures?
- What do you think could be the drawback using smart phone application to deliver oral health information?
- Do you have any suggestions for using smart phone to promote oral health?

### Part 3: Interview guide: dental providers sector

*Access to dental services*

- Do you provide routine dental treatment for women during pregnancy?
- What’s your experience of working with pregnant women? How do you feel about it?
- Do you find pregnant women mostly attend for check-ups, treatment, or emergencies?
- How do they hear about your service? Do you know how they get to the clinic?
- Do medical providers refer patients to you for prenatal oral health care? How does this work?

*Relationship between poor maternal oral health and child oral health outcomes*

- Do you think there are benefits of working with the mother/pregnant mother and then the baby/child? Either for the mother and/or their baby/child.
- Are you aware of any protocol, evidence, guidelines around the relationship between maternal oral health and child oral health outcomes? Clinical guidelines for caring for pregnant women?
- Have you noticed that pregnant women, have specific needs regarding their dental care? How do you respond to this?

*Information and resources*

- Do you require other information or resources to help explain oral health or service issues to people? What do you think would be useful?

*Service strengthening*

- How do you feel about the support you receive for providing dental care to underserved pregnant women?
- Are there any organizational changes that you think might help us to improve the care and outcomes for this group?

*Smart phone application use*

- Are you familiar with smart phone application?
- Do you know any smart phone application related to patients’ health during pregnancy?
- Will you use smart phone application to look for oral health information if there is one?
- What do you think using smart phone application to deliver education and updates to health care providers?
- What kind of information will you like to see on smart phone application regarding oral health, video, blog, pictures?
- What do you think could be the drawback using smart phone application to deliver oral health information?
- Do you have any suggestions for using smart phone to promote oral health?

## Abbreviations

PCP: Primary care physicians
CBPR: Community-based participatory research
IRB: Institutional Review Board
OBGYN: Obstetrician and gynecologist
